# The impact of gender equity in agriculture on nutritional status, diets, and household food security: a mixed-methods systematic review

**DOI:** 10.1136/bmjgh-2019-002173

**Published:** 2020-03-29

**Authors:** Helen Harris-Fry, Hayaan Nur, Bhavani Shankar, Giacomo Zanello, Chittur Srinivasan, Suneetha Kadiyala

**Affiliations:** 1 Department of Population Health, London School of Hygiene and Tropical Medicine, London, UK; 2 Centre for Development, Environment and Policy, SOAS University of London, London, UK; 3 School of Agriculture, Policy and Development, University of Reading - Whiteknights Campus, Reading, Berkshire, UK

**Keywords:** child health, maternal health, nutrition, systematic review

## Abstract

**Introduction:**

Undernutrition rates remain high in rural, low-income settings, where large, gender-based inequities persist. We hypothesised that increasing gender equity in agriculture could improve nutrition.

**Methods:**

We conducted a systematic review to assess the associations between gender-based inequities (in income, land, livestock, and workloads) and nutrition, diets and food security outcomes in agricultural contexts of low-income and middle-income countries. Between 9 March and 7 August 2018, we searched 18 databases and 14 journals, and contacted 27 experts. We included quantitative and qualitative literature from agricultural contexts in low-income and middle-income countries, with no date restriction. Outcomes were women’s and children’s anthropometric status, dietary quality and household food security. We conducted meta-analyses using random-effects models.

**Results:**

We identified 19 820 records, of which 34 studies (42 809 households) met the inclusion criteria. Most (22/25) quantitative studies had a high risk of bias, and qualitative evidence was of mixed quality. Income, land and livestock equity had heterogeneous associations with household food security and child anthropometric outcomes. Meta-analyses showed women’s share of household income earned (0.32, 95% CI −4.22 to 4.86; six results) and women’s share of land owned (2.72, 95% CI -0.52 to 5.96; three results) did not increase the percentage of household budget spent on food. Higher-quality studies showed more consistently positive associations between income equity and food security. Evidence is limited on other exposure–outcome pairings.

**Conclusions:**

We find heterogeneous associations between gender equity and household-level food security. High-quality research is needed to establish the impact of gender equity on nutrition outcomes across contexts.

**PROSPERO registration number:**

CRD42018093987.

**Key questions**

**What is already known?**

- Maternal and child undernutrition is most common in rural areas of low-income and middle-income countries, where large gender-based inequities in the agricultural sector co-occur.
- Reviews of the effects of women’s empowerment on nutrition outcomes show mixed results, but the relative effects of gender equity are poorly understood, both conceptually and empirically.

**What are the new findings?**

- The evidence base on the associations between gender equity in agriculture and nutrition, diets and household food security is limited and of mixed quality, with evidence on nutritional status and diet outcomes particularly lacking.
- Gender equity in terms of income, land and livestock has heterogeneous associations with household food security, although higher-quality studies show more consistently positive findings.

**What do the new findings imply?**

- High-quality quantitative and qualitative research is needed to establish the extent and processes by which gender equity affects nutrition, diets and food security outcomes across contexts.

## Introduction

Undernutrition among women and children remains a major public health problem in most low-income and middle-income countries, and is most prevalent in rural areas.^1^ Yet, estimates indicate that implementation of 10 nutrition interventions at 90% coverage would only reduce child stunting by 20%,^2^ half of the World Health Assembly target of a 40% reduction by 2025. New approaches are needed from other nutrition-relevant sectors such as agriculture.^2^

A well-functioning agriculture sector can improve nutrition outcomes through several pathways.^3^ For instance, farmers can increase the quantity and nutrient density of produce for consumption, generate more agricultural income to purchase nutritious foods,^4 5^ or increase availability and affordability of nutritious foods in markets.^3^ Many agricultural interventions developed to improve nutrition outcomes, such as promotion of biofortified foods, homestead food production and livestock keeping, have improved household and child dietary intakes.^5^

However, gender-based inequities within agriculture may limit the sector’s potential to provide nutritious diets and improve nutrition outcomes.^5^ In many low-income rural settings, women have lower ownership and use of land, livestock and other productive assets,^5^ lower economic participation and wage rates,^6^ and heavier workloads,^7^ compared with men.

Although it is plausible that closing the gender gap in agriculture could improve nutritional outcomes, this hypothesis remains conceptually underdeveloped and reliant on few, commonly cited studies^8–10^ or reviews^11 12^ of women’s empowerment, rather than relative measures of gender inequity. Several pathways may exist between gender inequities in agriculture and nutrition outcomes (online supplementary figure 1). One pathway may exist via women’s increased control over household spending decisions, leading to higher investment in women’s and children’s nutrition.^11^ Another pathway may be through improvements in household food availability.^13^ Studies indicate that agricultural production is more efficient in equitable households, leading to higher yields overall.^14^ At the societal level, gender equity could reduce poverty and improve nutritional outcomes^15^ through increases in economic equality between households.

10.1136/bmjgh-2019-002173.supp1 Supplementary data

Identifying the potential for equity-focused interventions to effectively complement existing nutrition and agricultural interventions requires better understanding of the effects of gender inequities in agriculture on nutritional outcomes. We systematically reviewed quantitative and qualitative evidence on the associations between gender inequities and women’s and children’s nutritional status, their diets, and household food security in agricultural settings of low-income and middle-income countries.

## Methods

### Search strategy and selection criteria

We conducted a mixed-methods systematic review of the associations between gender inequities in agriculture and women’s and children’s nutritional status, dietary quality and household food security. We followed Preferred Reporting Items for Systematic Reviews and Meta-Analyses guidelines (checklist in online supplementary table 1) and a prospectively registered protocol.

We included experimental and observational quantitative and qualitative literature, including peer-reviewed and grey literature, but not historical analyses. We included studies with original empirical evidence reporting a causal, correlational or descriptive link between at least one exposure and outcome. We did not exclude studies based on language or publication date. We included studies from agricultural settings (at least half of the study population containing at least one household member involved in United Nations-defined ‘agriculture’^16^) in low-income and middle-income countries (categorised using World Bank classifications).^17^

Our exposures focused on male–female disparities in rural agriculture settings in low-income and middle-income countries. The exposures we included were

- Income, including wage rates, workforce participation and labour market opportunities, but not including decision-making about the use of income.
- Land and livestock, including inheritance rights, statutory and usufruct ownership, and access or use, but not including decision-making about the use of produce generated from land or livestock.
- Workloads, including hours worked, effort or physical activity levels, but not including decision-making about time use or the division of labour.

We anticipated that studies would rarely specify what proportion of income, land or work effort was allocated to or spent on agricultural versus non-agricultural activities. Consequently, our exposures pertained to all income, land, livestock and workloads within agricultural contexts, rather than that dedicated to agricultural work specifically.

Prespecified outcomes related to women’s (aged 15–49 years) and children’s (aged under 5 years) nutritional status and dietary quality, as well as overall household food security.

- Child nutrition outcomes were underweight (mean or low (<−2 SD) weight-for-age), wasting (mean or low (<−2 SD) weight-for-height) or stunting (mean or low (<−2 SD) height-for-age).
- Women’s nutritional status was indicated by mean or low (<18.5) body mass index (kg/m^2^).
- Dietary quality was defined as the Minimum Dietary Diversity for Women^18^ or older versions of this score, WHO Infant and Young Child Feeding indicators^19^ or any measure of dietary adequacy that accounts for nutritional requirements.
- Household food security indicators were household food expenditures, percentage share of household budget spent on food (‘food share’), percentage share of food budget spent on staple foods, household dietary diversity (count of food groups consumed by the household) or the Household Food Insecurity Access Scale.^20^

In the qualitative literature, nutrition, diets or food security outcomes described more broadly were allowed.

Between 9 March and 7 August 2018, we searched the following databases: EBSCO, Medline, Scopus, Web of Science, Popline, CAB, Eldis, OpenTrials, Bridge Data and AGRIS. An example of a full search string is in online supplementary table 2, using synonyms for each term in the following structure: [(land OR livestock OR income OR workload) AND (diets OR nutrition OR food security) AND (gender) AND (low-income or middle-income countries)]. We hand-searched eight repositories and 14 journals (listed in online supplementary table 3), plus references lists of relevant publications, and contacted 27 experts. Identified papers were exported into EPPI-Reviewer V.4 systematic review software and papers were doubly screened by HN and HH-F. Any disagreements were resolved by SK. Data were extracted into pretested forms by HN and were checked by another reviewer.

### Data analysis

We extracted coefficients and their measures of variance, the gender equity gap (differential in exposure between men and women), and qualitative quotes and conclusions. We contacted authors if information on equity gap was missing. We also extracted author names, country, dates of study and publication, study design, analysis method, sample size and response rate. When there was more than one publication on the same association using the same data (n=5), we prioritised peer-reviewed reports. When we found multiple associations for the same exposure–outcome pair within one study (n=14), such as studies reporting sensitivity analyses or reporting both crude and adjusted models, we extracted the coefficients of the main result reported by the author.

We extracted any results on the following preplanned intermediate outcomes: agricultural production, household income, women’s empowerment, household poverty and economic inequity between households. We also looked for information on climatic or environmental mediating factors influencing our exposures or influencing the relationship between exposures and outcomes.

Two reviewers independently coded risk of bias and study quality. We assessed risk of bias in quantitative literature using an adapted version of the Risk of Bias in Non-randomised Studies of Interventions (ROBINS-I) tool.^21^ This assesses bias due to confounding, sample selection, exposure classification, missing data, outcome measurement and outcome reporting. We excluded the domain on deviation from intended interventions and added a domain on instrumental variables (specifically, if there was evidence that the exclusion restriction did not hold and the instrument was relevant). To assess risk of bias due to confounding, we preidentified the following confounders as relevant to most studies: household income, poverty or economic status; land ownership; household size or composition; caste/ethnicity/religion; and household attitudes towards gender equity. Following the ROBINS-I guidance, we also evaluated confounding in each study by identifying other confounders relevant to each setting or particular study, plus other confounders that the study authors identified as potentially important. Each coefficient was categorised as ‘low’, ‘moderate’, ‘serious’ or ‘critical’ risk of bias based on the assessment of risk of bias in all domains.

Quality of qualitative literature was assessed across 11 domains, using the Lockwood, Munn and Porritt tool.^22^ This tool assesses appropriateness of research methodology, sampling, data collection, representation and analysis of data, interpretation of results, conclusions and ethics. It also assesses researchers’ own evaluation of their influence and positionality in relation to the research. Each study is given an overall assessment of ‘high’, ‘medium’, ‘low’ or ‘critical’ quality.

We did not exclude any studies based on risk of bias or study quality. We originally planned to exclude critical-quality qualitative studies but have included all to be consistent in our treatment of quantitative and qualitative evidence. This resulted in the inclusion of one more qualitative study that did not change overall findings.

The counterfactual for the exposures was no change in gender equity. In some studies, the gender equity exposure was a ratio or difference between women versus men, or women versus the household (women plus men). In other studies, men and women entered the regression as two separate exposures by estimating, for example, the effects of women’s land ownership controlling for men’s land ownership. When modelled separately, we calculated the impact of gender equity by subtracting one coefficient from the other (eg, by subtracting the coefficient of men’s land ownership on the outcome from the coefficient of women’s land ownership on the outcome).

Due to differences in scales of exposures between studies (like currencies or units of land) and differences in the size and direction of gender equity, we standardised the estimates. First, we calculated the ‘equity gap’ (difference between baseline or mean level of exposure and equity). Then, we scaled the estimates by the equity gap to represent the proportion of the male–female difference represented rather than the absolute difference. The standardised effect size can be interpreted as the average marginal effect of approaching equity.

In cases where the exposure compared women with men, the equity gap was calculated as half of the difference between men and women. For example, a study from Niger found that predicted male income was 149 336 Franc Communauté Financière Africaine (FCFA) and female income was 57 720 FCFA, so the equity gap was 45 808 FCFA.^23^ In cases where the exposure compared women with the household, the equity gap was the difference between women and 0.5 (where 0.5 indicates an equal split between men and women). For example, a study from Cote d’Ivoire reported women’s share of household income as 0.20, so the equity gap was 0.30.^10^

To conduct a meta-analysis, we needed more than one study per exposure–outcome pair with variance estimates, and analysis methods that allow pooling of results. The standardisation of coefficients was intended to allow pooling of differently measured exposures within income, land and workload domains, but in practice, the studies included in meta-analyses used the same exposures. We conducted meta-analyses with the ‘admetan’ command in SSTATA V.SE 14.2 using random-effects models. We report *I*^2^ to show the variation attributable to heterogeneity and τ^2^ to describe between-study variance. Results from other studies that could not be included in meta-analysis are narratively described. We first use all available evidence and then describe sensitivity to risk of bias where possible.

Planned additional analyses aimed to identify geographical variation, intermediate outcomes and evidence of environmental influences on these effects. These secondary aims and the inclusion of qualitative evidence were intended to explain variance in results and avoid doing a ‘black box’ review of limited policy relevance.

## Results

**Figure 1:**
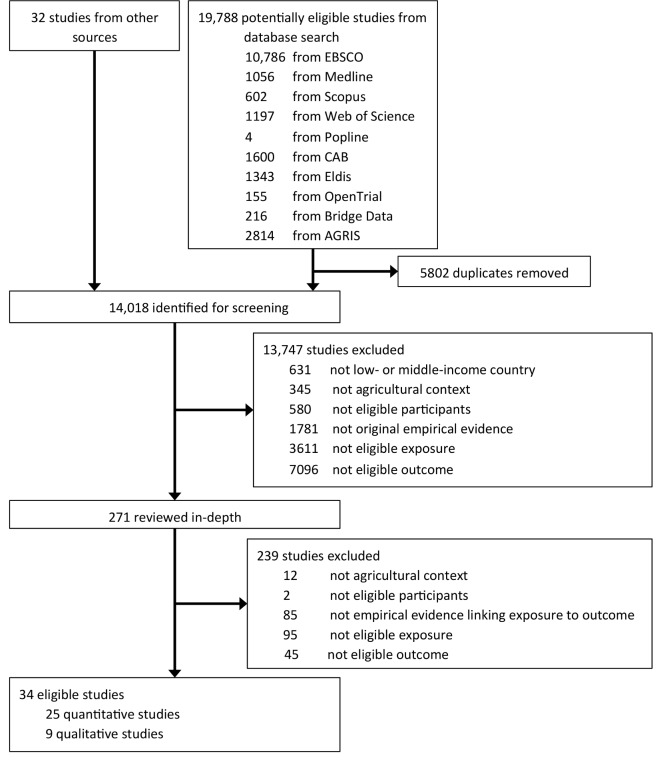
Flowchart of the study selection process.

We screened 19 820 studies and included 34 for analysis, as shown in figure 1. In the quantitative literature we found 25 studies from 24 publications containing 39 results.^10 23–45^ Against protocol, we included a working paper by Senauer and Garcia,^27^ rather than the journal article,^46^ because it used more waves of a panel and it reported on more relevant outcomes. In the qualitative literature, we found nine studies from seven publications.^47–53^

Characteristics of included studies are given in online supplementary table 4. Most evidence came from sub-Saharan Africa (n=20, 34%), followed by South Asia (n=7, 21%), East Asia and Pacific (n=5, 15%) and Latin America (n=2, 6%). Publication dates for the quantitative studies ranged from 1988 to 2018, whereas all qualitative studies were published in the last decade.

In the quantitative studies, the most common exposure was gender inequities in income, and the most common outcome was share of household expenditures spent on food (food share). No studies reported on women’s nutritional status or their dietary quality. Most quantitative studies carried a high risk of bias, ranging from moderate (n=4) to critical (n=16) (online supplementary table 5) mainly because the exposures are difficult to experimentally manipulate, so most studies applied econometric causal inference methods to observational data or reported correlational evidence. Instrumental variables were often assessed as inadequate; fixed-effects models often failed to account for unobserved; time-varying confounding; and risk of misclassifying exposures was also common.

In qualitative literature, the most common exposures were income and workloads (both n=5), and the most common outcome was household food security (n=5). Quality ranged from critical (n=1) to high (n=2) (online supplementary table 6). Common limitations were the lack of methodological detail on analysis and interpretation of results, and lack of consideration of bias introduced by the researchers’ influence. Quality of quantitative and qualitative evidence is not comparable because we used different appraisal tools.

### Gender equity in earned income, wage rates and workforce participation

**Figure 2:**
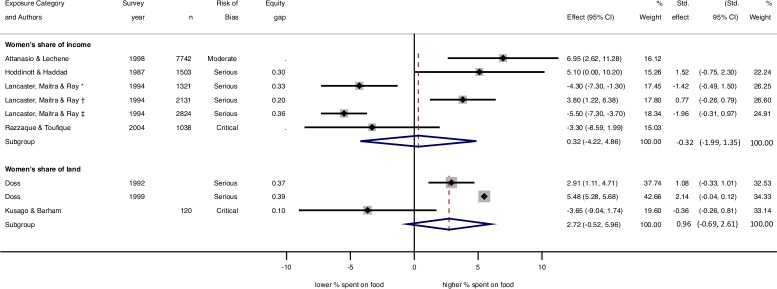
Forest plot of effects of women’s share of household income and women’s share of household land on percentage share of household expenditures spent on food. Weights are from random-effects models. Equity gap was calculated as the difference between perfect equity (0.5) and women’s proportion of income or land. *I*^2^ for women’s share of income=91.7% unstandardised effects; 94.2% standardised effects. *I*^2^ for women’s share of land=89.3% unstandardised effects; 97.8% standardised effects. *, rural Kerala; †, rural Maharashtra; ‡, rural Bihar; Std., standardised.

Twelve quantitative studies with varied risk of bias reported associations between gender equity in income and household food security, where food security was measured as food share, (log) food expenditure and Household Food Insecurity Access Scale. Results were highly heterogeneous (table 1 and figure 2).

**Table 1:** Associations between gender equity in income and food security, diets and nutritional status

	Exposure	Analysis	Year	N	Equity gap	Effect	95% CI	Standardised effect	Standardised 95% CI
Outcome: food share (not included in meta-analysis)									
Lachaud^31^	Women’s share of household income	2-stage tobit	1994	4744	0.26*	2.36	0.25 to 4.47	0.62	0.07 to 1.18
McCarthy and Kilic^35^	Women earn all unpooled income versus men earn all	SUR	2010–2013	3858	0.06†	0.70	−1.46 to 2.85	0.00	−0.00 to 0.00
Outcome: Ln (food expenditure)									
Hopkins *et al*^23^	Women’s income versus men’s	2SLS	1990	452	45 808†	0.00	–	0.01	–
Duflo and Udry^29^	Change in women’s income versus change in men’s	2SLS	1985–1988	973	0.71†	0.17	–	0.12	–
Josephson^34^	Change in women's Ln agricultural income versus men's	DiD, IV	2010–2013	693	0.89†	0.15	–	0.13	–
Aromolaran^28^	Women’s share of household income	2SLS	1999–2000	2573	0.20*	−0.13	−0.23 to −0.03	−0.03	−0.05 to −0.01
Outcome: food expenditure									
McCarthy and Kilic^35^	Women earn all unpooled income versus men earn all	OLS FE	2010–2013	3858	0.06†	0.06	−0.04 to 0.17	0.03	−0.00 to 0.00
Outcome: Household Food Insecurity Access Scale									
Van den Broeck *et al*^30^	Women’s employment in agricultural export sector versus men’s	DiD	2013–2016	461	−0.07‡	−0.15	–	0.01	–
Outcome: child’s energy adequacy ratio (intakes/requirements)									
Senauer and Garcia^27^	Mother’s wage versus father’s	2SLS	1983–1984	2320	0.01†	0.07	–	0.00	–
Outcome: height-for-age z-score									
Senauer and Garcia^27^	Mother’s wage versus father’s	2SLS	1983–1984	2320	0.01†	0.38	–	0.00	–
Marinda^32^	Mothers’ income minus men’s	2SLS	2003	129	–	−0.00	–	–	–
Outcome: low height-for-age									
Gaiha and Kulkarni^25^	Male–female wage difference	Poisson	1994	26 854	1.06†	−0.07	−0.01 to −0.13	−0.08	−0.14 to −0.01
Lachaud^31^	Women’s share of household income	2-stage probit	1994	1352	0.28*	−0.23	−0.43 to −0.03	−0.03	−0.06 to −0.00
Outcome: weight-for-height z-score									
Senauer and Garcia^27^	Mother’s wage versus father’s	2SLS	1983–1984	2320	0.01†	−0.19	–	−0.00	–
Outcome: low weight-for height									
Lachaud^31^	Women’s share of household income	Probit	1994	1352	0.28*	−0.09	−0.43 to 0.25	−0.00	−0.01 to 0.01
Outcome: weight-for-age z-score									
Shoo^33^	Mother has a non-farming source of income versus father	OLS	2011	152	–	−0.04	–	–	–
Outcome: low weight-for-age									
Lachaud^31^	Women’s share of household income	Probit	1994	1352	0.28*	−0.27	−0.47 to −0.07	−0.04	−0.06 to −0.01

*Difference between observed level of exposure and perfect equity, defined as 0.5.

†Half of the difference between men and women.

‡Calculated in a Bayesian combination as the difference in probability that a man versus a woman works in the horticultural export sector.

DiD, difference-in-difference; FE, fixed effects; IV, instrumental variable; Ln, Natural logarithm; OLS, ordinary least squares; 2SLS, two-stage least squares; SUR, seemingly unrelated panel regression.

Eight studies reported associations between income equity and household food share. Positive associations were observed in Mexico,^45^ Cote d'Ivoire^10^ and rural Maharashtra (India)^26^; null results in Bangladesh,^24^ Malawi^35^ and Burkina Faso^31^; and negative associations in rural Kerala and Bihar (India).^26^ Consistent with this, a meta-analysis of six studies (excluding two due to their different analysis methods) showed overall null (0.3 percentage points, 95% CI −4.2 to 4.9), highly heterogeneous (*I*^2^ 91.7%, τ^2^ 28.5) unstandardised results for women’s relative income on the share of household budget spent on food (figure 2). Excluding a further two studies due to lack of information provided to calculate an equity gap, a meta-analysis of the four standardised results also showed null findings (−0.32, 95% CI −1.99 to 1.35) (figure 2).

Five studies, all from sub-Saharan Africa, measured food security in terms of total food expenditures. Positive associations were observed in Cote d’Ivoire^29^ and one study from Malawi,^34^ whereas another study from Malawi^35^ and Niger^23^found no association, and a study from Nigeria found a negative result.^28^ We could not pool estimates due to lack of variance measures. Of note, the study from Niger also found that the timing of men’s income flows had no impact on expenditure, whereas the timing of women’s income flows did affect food expenditures.^23^

A sensitivity analysis of all 12 studies on household food security indicates that results are sensitive to risk of bias, with the three studies carrying the lowest risk of bias (from Mexico,^45^ Cote d’Ivoire^29^ and Senegal^30^), showing that increasing women’s relative income or employment increased food security. In Mexico, an increase in women’s share of income of one percentage point was associated with 7.0 percentage points (95% CI 2.62 to 11.28) higher food budget share.^45^ To minimise risk of confounding, the authors used a randomised cash transfer programme (Progressa) as an instrument for women’s income share. In a study from Cote d’Ivoire, where men and women often farm different crops on separate plots, the authors used rainfall shocks to instrument income changes for men and women. A 10% increase in income from female-controlled crops was associated with a 2% increase in food consumption, whereas the same increase in income from male-controlled crops gave a smaller increase in food consumption of 0.6%.^29^ Finally, in Senegal, women’s employment in the horticultural export sector was positively associated with households’ perceived food security (Household Food Insecurity Access Scale): households in which any women were employed had 11.3% lower probability of food insecurity, whereas male employment gave null results.^30^ However, women had a higher probability of being employed, so closing the gender gap would decrease food security.

Only one study reported associations between income equity and children’s diets, showing that neither mothers’ nor fathers’ predicted wages were associated with their preschoolers’ calorie adequacy ratio in the rural Philippines.^27^

Five studies reported child anthropometric outcomes. Four studies reported associations between income equity and height-for-age, with studies from India^25^ and Burkina Faso^31^ showing positive associations (lower prevalence of low height-for-age) and another two from the Philippines^27^ and Kenya^32^ showing no association with mean height-for-age. Two studies from the Philippines^27^ and Burkina Faso^31^ showed no association with weight-for-height. For weight-for-age, a Tanzanian study showed a negative association,^33^ but the Burkina Faso study showed a positive association (lower prevalence of low weight-for-age).^31^ Results could not be pooled due to different analysis methods and lack of variance estimates, and we could not assess sensitivity to risk of bias because they were all rated critical.

Qualitative literature indicated that relative earned income was an important determinant of household food security^51 53^ and dietary quality,^49^ but that gender norms determining participation in income-generating activities limited this. Findings agreed within and between studies. In Malawi,^51^ Tanzania and Nicaragua,^53^there was a common perception that men spent too much of their income on personal expenses, whereas women’s income was used for the household and to buffer against shocks to income, thereby (the authors argue) improving food security.^53^ However, gender norms determining participation in income-generating activities were found to suppress this. For example, a Malawian study reported that gender norms determining the participation in crop sales (such as lack of mobility inhibiting women from travelling to more lucrative markets) limited women’s earned income. Those who did earn income customarily handed it over to men.^53^

In the African studies, respondents placed emphasis on conflict between men’s and women’s overspending decisions, and perceptions that men were spending the money irresponsibly. In contrast, respondents from a Nepali study focused more on their perceptions of duty and deference to their husbands, and explanations that men who earn income deserve larger allocations of food, as described by one female respondent: ‘They will only earn and bring the money if they have fulfilled their appetite. They will only be able to work if they eat properly’ (Morrison *et al*, p380)[49].

### Gender equity in ownership and use of land

Nine studies (11 results) reported associations between land and/or livestock equity on household food security (table 2 and figure 2), including seven studies on equity in land,^36–38 40–43^ one on equity in livestock^39^ and another on a combined exposure of land and livestock.^38^

**Table 2:** Associations between gender equity in land and/or livestock and food security, diets and nutritional status

	Exposure	Analysis	Years	N	Equity gap	Effect	95% CI	Standardised effect	Standardised 95% CI
Outcome: food share (not included in meta-analysis)									
Menon *et al*^37^	Women have land use certificate versus men	OLS FE	2004–2008	14 826	0.19*	0.60	–	0.11	–
Muchomba^41^	Joint land titling versus men only	DiD	1994–2009	1061	1.00†	0.10‡	–	0.10	–
Quisumbing and Maluccio^38^	Ln wife's land size at marriage versus husband's, in hectares	2SLS	–	114	0.24*	8.40	–	2.01	–
Quisumbing and Maluccio^38^	Ln value of wife's land and livestock at marriage versus husband's, in Ethiopian birr	2SLS	1997	1347	0.85*	642.3	–	545.8	–
Pangaribowo^39^	Women’s share of household livestock assets	OLS	1997–2007	–	–	−15.2	–	–	–
Outcome: Ln (food expenditure)									
Muchomba^41^	Joint land titling versus men only	DiD	1994–2009	1061	1.00†	0.43‡	–	0.43	–
Outcome: Ln (household food diversity count)									
Kumar^42^	Share of household land size farmed by women (jointly or individually)	OLS	1986	213	−0.10§	0.18	0.10 to 0.27	−0.02	−0.03 to −0.01
Outcome: household dietary diversity score									
Santos *et al*^36^	Women’s name on land title versus men’s	PSM and OLS	2012	1035	0.25†	−0.06	−0.26 to 0.14	−0.02	−0.06 to 0.03
Outcome: height-for-age z-score									
Jin and Iannotti^44^	Women’s livestock value (solely or jointly owned) minus men’s (solely owned), in Kenyan shillings	OLS	2010	183	1.26†	0.10	–	0.13	–
Outcome: weight-for-height z-score									
Jin and Iannotti^44^	Women’s livestock value (solely or jointly owned) minus men’s (solely owned), in Kenyan shillings	OLS	2010	183	1.26†	0.01	–	0.01	–
Outcome: weight-for-age z-score									
Jin and Iannotti^44^	Women’s livestock value (solely or jointly owned) minus men’s (solely owned), in Kenyan shillings	OLS	2010	183	1.26†	0.05	–	0.06	–

*Half of the difference between men and women.

†Assuming all assets should be jointly owned.

‡Combined effects on budget spent on homegrown foods and market-bought foods.

§Difference between observed level of exposure and perfect equity, defined as 0.5.

DiD, difference-in-difference; FE, fixed effects; Ln, Natural logarithm; OLS, ordinary least squares; PSM, propensity score matching; 2SLS, two-stage least squares.

Six results related gender equity in land ownership to food share. Two were positive, with a three to five percentage point increase in food share from a one percentage point increase in women’s share of household agricultural land in Ghana.^43^ The other four results—women’s share of land in Malaysia,^40^ women’s versus men’s land at marriage in an Indonesian matrilineal context,^38^ women’s versus men’s land use certificates in Vietnam^37^ and joint land titling in Ethiopia^41^—were null or very small (<1% difference). A meta-analysis of the three unstandardised results that reported variance estimates (figure 2) showed overall null findings (2.72, 95% CI −0.52 to 5.96) and high heterogeneity (*I*^2^ 89.3%, τ^2^ 6.4). Meta-analysis of the standardised results was also null (0.96, 95% CI −0.69 to 2.61).

One study reported associations between equity in land and food expenditure, showing no differences in food expenditure between households where land was held jointly by men and women in Ethiopia, compared with head-only land certification.^41^

Two studies reported associations between equity in land and household dietary diversity, showing mixed results. In India, women’s name on land title (compared with men’s) was not associated with household dietary diversity.^36^ In Zambia, a one-unit increase in the proportion of household land farmed by women was associated with 20% higher dietary diversity.^42^ However, women farmed a larger proportion of land than men, so increasing equity by reducing the share of land farmed by women would lower dietary diversity.^42^

The single study reporting on gender equity in livestock found that increasing women’s share of household livestock assets in Indonesia was associated with a lower food share (15 percentage points, no variance estimate),^39^ whereas the study that combined land and livestock into one exposure found very large increases in food share with increasing equity in Ethiopia. The authors found that decreasing husbands’ land and livestock assets by 10% and increasing wives’ assets by 10% would increase the food share by 64 percentage points.^38^

Out of the 11 results on land and/or livestock equity on food security, the two with comparatively lower risk of bias—both investigating effects of land titling—showed positive associations with food security.^37 41^ The results on land and livestock equity from other studies (all rated critical risk of bias) were more mixed.

No studies reported associations between land or livestock equity and maternal or child diets, or maternal body mass index. We found one study on child nutritional status, which was from Kenya. The value of women’s livestock ownership was positively associated with height-for-age and weight-for-age z-scores, but not weight-for-height, whereas men’s livestock was not associated with any anthropometric indicators.^44^

The three qualitative studies on gender inequities in land or livestock were from Malawi,^51^ Tanzania^53^ and Ethiopia,^53^ and they all described relationships with food security rather than diets or nutritional status. They found that, while gender equity could improve food security, this was likely constrained by other ways that men control decisions. For example, a Malawian study on matrilineal land inheritance reported that, although women owned land, men controlled decisions about how the land was used.^51^ In Tanzania and Ethiopia, women expressed similar concerns about their lack of control over decisions about livestock management and income generation.^53^

### Gender equity in time use and workloads

Only one quantitative study, rated critical risk of bias, reported on gender inequity in time use. The gender gap was large: women in Zambia spent around 621 hours/year more than men on household maintenance.^42^ Compared with men, women’s time spent on household maintenance had smaller associations with household dietary diversity, although neither effect was statistically significant.^42^

Workload was a common theme in the qualitative literature. Women’s comparatively higher work burdens and lack of household support from men for childcare and cooking were linked to poorer diets for women and children. For example, in India, women’s higher work burdens in the fields came at the expense of their time for cooking and eating sufficient food, as described by one village woman: ‘The women keep working the whole day from early morning to late night, and if she is not feeling hungry then she won’t eat and she will go to bed. Then early morning she again will start working and if they not having food properly then that is why they get sick’ (Nichols, p1415).[48] Women’s comparatively higher workloads also added to their anxiety and lack of appetite.^48^

Nepalese women reported that they ate less if they did not work outside of the home. This extended to differences in the allocation of foods within women too: ‘*My sisters-in-law do lots of work within the home as well as outside the home, so I give them more food. They bring grasses, husks and firewood. I only cook food’* (Morrison *et al*, p380)[49].

A lack of spousal support was also identified as limiting women’s time available to provide for their children’s nutrition, as summarised by a mother from rural Gambia: ‘*They* (husbands) *should be helping us but unfortunately they are not doing it. What can one do when a man says no!’* (Mwangome *et al*, p169)[50].

### Effects of gender equity on intermediate outcomes

None of the included studies reported on agricultural production, household poverty, or economic inequity. Two studies reported on indicators of household income, showing mixed results (one positive^29^ and one null,^35^ and another study reported positive associations between land equity and indicators of women’s empowerment^36^ (table 3). We found no information on climatic or environmental influences.

**Table 3:** Associations between gender equity and hypothesised intermediate outcomes

	Exposure	Outcome	Analysis	Years	N	Equity gap	Effect	95% CI	Standardised effect	Standardised 95% CI
Gender inequity in income→household income										
Duflo and Udry^29^	Predicted change in women’s income versus predicted change in men’s	Ln total expenditure	2SLS	1985–1988	973	0.71*	0.18	–	0.13	–
McCarthy and Kilic^35^	Female earn all unpooled income versus men earn all	Total consumption expenditures per capita	OLS FE	2010–2013	3858	0.06*	0.05	−0.03 to 0.13	0.00	0.00 to 0.00
Gender inequity in land or livestock→women’s empowerment										
Santos *et al*^36^	Women's name on land title (solely or jointly) versus men's name only	Women take decisions about whether to take a loan from a Self Help Group or microfinance institution	PSM and OLS	2012	1035	0.25†	0.14	0.08 to 0.20	0.04	0.02 to 0.05
Santos *et al*^36^	Women's name on land title (solely or jointly) versus men's name only	Women take decisions about purchase of productive assets	PSM and OLS	2012	1035	0.25†	0.15	0.07 to 0.23	0.04	0.02 to 0.06
Santos *et al*^36^	Women's name on land title (solely or jointly) versus men's name only	Women take decisions about food purchase and consumption decisions	PSM and OLS	2012	1035	0.25†	0.13	0.05 to 0.21	0.03	0.01 to 0.05
Santos *et al*^36^	Women's name on land title (solely or jointly) versus men's name only	Women take decisions about how to use the plot of land	PSM and OLS	2012	1035	0.25†	0.13	−0.01 to 0.27	0.03	0.00 to 0.07

*Half of the difference between men and women.

†Assuming all assets should be jointly owned.

FE, fixed effects; Ln, Natural logarithm; OLS, ordinary least squares; PSM, propensity score matching; 2SLS, two-stage least squares.

### Publication bias

Four studies were published as working papers (one rated ‘moderate’ and three rated ‘critical’ risk of bias), two were dissertations (both rated ‘critical’ risk of bias), and the rest were peer-reviewed articles. We do not report funnel plots or Eggar tests because of an insufficient number of studies per exposure-outcome pairing.^54^

## Discussion

Gender equity in income, land, and livestock ownership has heterogeneous associations with household food security in agricultural settings. Quality of evidence is considered low due to high risk of bias and lack of variance estimates, but results indicate more, positive associations between equity (in income and land ownership) and household food security in higher-quality studies. Qualitative studies suggest that impacts of gender equity in agriculture on food security may be suppressed by women’s comparatively lower control over income or agricultural production processes. We lack evidence on workload equity, and maternal and child diets and nutritional status.

The heterogeneity in results may reflect regional or temporal variance, including different pathways to impact, or differences in study design, measurement, and analysis methods. Other reviews on the nutritional effects of women’s empowerment^11 55^ and women’s time use^56^ also find varied results, and also point to measurement challenges as a possible explanation. Accurate measurement of gender equity is challenging, and may not always capture women’s relative control over these resources. For example, women may have a land deed but not control decisions about the use of that land. This review highlights the need to develop more robustly measurable indicators of gender equity, and the need to test hypothesised pathways from gender equity to food security and nutrition outcomes.

Our research question is difficult to answer with randomised study designs because the societal structures underpinning gender gaps are difficult to experimentally manipulate and may take generations to change. Therefore, more advanced methods for causal identification are required to estimate the effects of gender inequities on nutrition outcomes, beyond linear regressions. As shown by Attanasio and Lechene,^45^ different analysis methods will yield different results, illustrating the extent to which poor causal identification may compromise our conclusions. Many studies in our review employed various analytical methods to draw causal inference using observational data, and those using weaker methods, with high risk of confounding, may have compromised our conclusions.^45^New research with robust alternative causal inference methods such as quasi-experimental designs, better data and metrics on gender equity, and increasing use of randomised field trials in agriculture-nutrition interventions, may provide more consistent results.

### Strengths and limitations

Our review benefitted from a systematic approach, wide search, and duplicate assessment of study inclusion, risk of bias, and quality. However, our ability to conduct meta-analyses was limited by the heterogeneity of exposure-outcome pairings retrieved, and we found scarce evidence from Latin America, on maternal and child diets, and on gender equity in time use. This constrained us from conducting a planned sub-group analysis by region.

Publication bias and reporting bias are possible limitations, although the convention of publishing working papers and conference papers in the social sciences may reduce this risk. No studies registered protocols or analysis plans. These procedures, standard practice in medical trials, are not yet commonplace in the social sciences – one example of the challenges of assessing risk of bias in mixed-methods, interdisciplinary reviews.

Our selection of exposures restricts our review to a limited set of structural inequities. As indicated by the qualitative results, gender inequity in decision-making and control may mediate effects on nutrition. Careful qualitative work to understand barriers to overcoming inequities and pathways between inequities and nutrition outcomes is needed.

The ROBINS-I tool that we used is designed to appraise experiments in the biomedical sciences; we found it less appropriate for social science studies that use other causal inference methods. Also, because outcomes were not specified *a priori* (a convention not yet widely adopted outside of the biomedical sciences), some highly prominent, otherwise high-quality economic studies were classified as ‘moderate’ risk of bias.^29 45^ Quality assessment tools for multi-disciplinary reviews are needed.

## Conclusions

There is limited evidence that closing the gender gap in agriculture will improve nutrition outcomes or dietary quality. Many potential policies could address gender inequities in agriculture, including laws to close gender wage gaps, women’s land titling schemes, and equitable land inheritance laws.^37^ Agricultural interventions could also be designed to redress inequities in agriculture, by ensuring that they do not disproportionately burden women and/or benefit senior men.^57^ This requires in-depth understanding of gender dynamics in agriculture, for different rural livelihood strategies, including gender differences in decision-making, workload, and access to inputs, services, markets, and social support. Beyond possible food security and nutritional benefits, such approaches are also worthwhile for reducing poverty^57^ and improving well-being, if gender equity is considered a normative goal in its own right.
