# Supplementary material for: The impact of gender equity in agriculture on nutritional status, diets, and household food security: a mixed-methods systematic review
Source: BMJ Glob Health. 2020 Mar 29;5(3):e002173. doi: 10.1136/bmjgh-2019-002173 (PMC7170429; doi:10.1136/bmjgh-2019-002173)
Supplement: Supplementary data [file bmjgh-2019-002173supp001.pdf]

Helen A Harris-Fry *et al.* | Online Supplementary Material

Supplemental file: Effects of gender inequities in agriculture on food security, diets and nutritional status: a mixed-methods systematic review

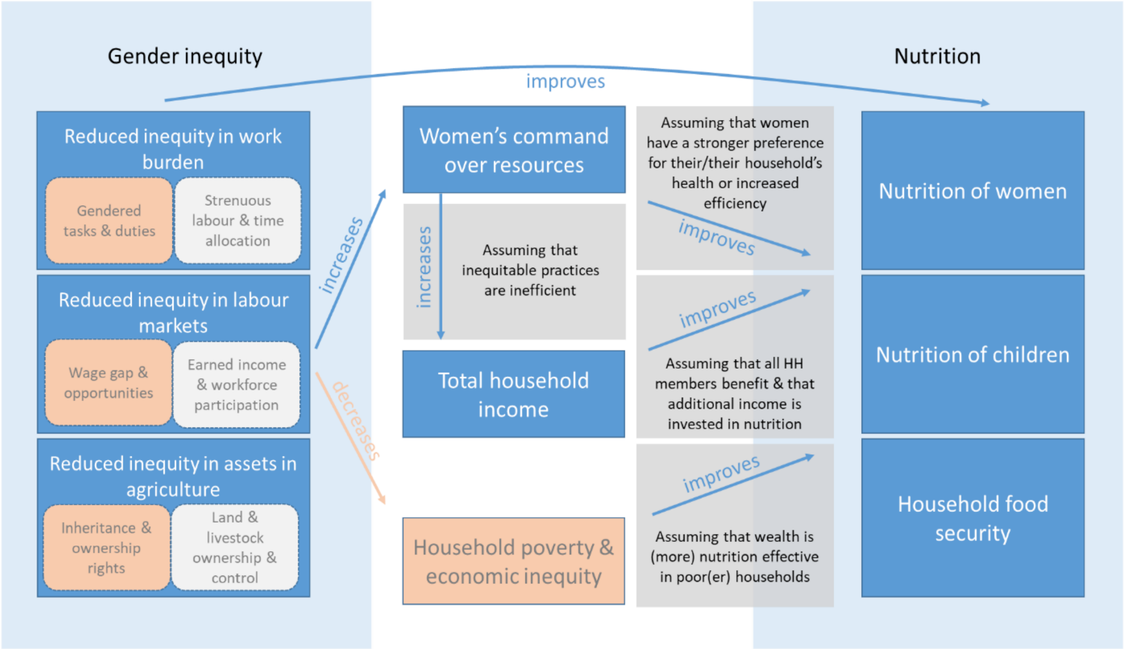

Supplemental Figure 1: Hypothesised pathways from gender equity to improved nutrition outcomes

Helen A Harris-Fry *et al*. | Online Supplementary Material

Supplemental Table 1: PRISMA checklist

**Section/topic**	**#**	**Checklist item**	**Reported on page #**
**Title**			
Title	1	Identify the report as a systematic review, meta-analysis, or both.	1
**Abstract**			
Structured summary	2	Provide a structured summary including, as applicable: background; objectives; data sources; study eligibility criteria, participants, and interventions; study appraisal and synthesis methods; results; limitations; conclusions and implications of key findings; systematic review registration number.	3
**Introduction**			
Rationale	3	Describe the rationale for the review in the context of what is already known.	5
Objectives	4	Provide an explicit statement of questions being addressed with reference to participants, interventions, comparisons, outcomes, and study design (PICOS).	6
**Methods**			
Protocol and registration	5	Indicate if a review protocol exists, if and where it can be accessed (e.g., Web address), and, if available, provide registration information including registration number.	6
Eligibility criteria	6	Specify study characteristics (e.g., PICOS, length of follow-up) and report characteristics (e.g., years considered, language, publication status) used as criteria for eligibility, giving rationale.	6-8
Information sources	7	Describe all information sources (e.g., databases with dates of coverage, contact with study authors to identify additional studies) in the search and date last searched.	8<br>Suppleme ntal Table 3
Search	8	Present full electronic search strategy for at least one database, including any limits used, such that it could be repeated.	Suppleme ntal Table 2
Study selection	9	State the process for selecting studies (i.e., screening, eligibility, included in systematic review, and, if applicable, included in the meta-analysis).	8
Data collection process	10	Describe method of data extraction from reports (e.g., piloted forms, independently, in duplicate) and any processes for obtaining and confirming data from investigators.	8
Data items	11	List and define all variables for which data were sought (e.g., PICOS, funding sources) and any assumptions and simplifications made.	8
Risk of bias in individual studies	12	Describe methods used for assessing risk of bias of individual studies (including specification of whether this was done at the study or outcome level), and how this information is to be used in any data synthesis.	9
Summary measures	13	State the principal summary measures (e.g., risk ratio, difference in means).	10
Synthesis of results	14	Describe the methods of handling data and combining results of studies, if done, including measures of consistency (e.g., I^2) for each meta-analysis.	11

Helen A Harris-Fry *et al*. | Online Supplementary Material

Risk of bias across studies	15	Specify any assessment of risk of bias that may affect the cumulative evidence (e.g., publication bias, selective reporting within studies).	11
Additional analyses	16	Describe methods of additional analyses (e.g., sensitivity or subgroup analyses, meta-regression), if done, indicating which were pre-specified.	11
**Results**			
Study selection	17	Give numbers of studies screened, assessed for eligibility, and included in the review, with reasons for exclusions at each stage, ideally with a flow diagram.	Fig 1
Study characteristics	18	For each study, present characteristics for which data were extracted (e.g., study size, PICOS, follow-up period) and provide the citations.	Suppleme ntal Table 4
Risk of bias within studies	19	Present data on risk of bias of each study and, if available, any outcome level assessment (see item 12).	Suppleme ntal Tables 5 & 6
Results of individual studies	20	For all outcomes considered (benefits or harms), present, for each study: (a) simple summary data for each intervention group (b) effect estimates and confidence intervals, ideally with a forest plot.	Tables 1-3 & Fig 2
Synthesis of results	21	Present results of each meta-analysis done, including confidence intervals and measures of consistency.	Fig 2
Risk of bias across studies	22	Present results of any assessment of risk of bias across studies (see Item 15).	Suppleme ntal Tables 5 & 6
Additional analysis	23	Give results of additional analyses, if done (e.g., sensitivity or subgroup analyses, meta-regression [see Item 16]).	15, 21 & Table 3
**Discussion**			
Summary of evidence	24	Summarize the main findings including the strength of evidence for each main outcome; consider their relevance to key groups (e.g., healthcare providers, users, and policy makers).	24
Limitations	25	Discuss limitations at study and outcome level (e.g., risk of bias), and at review-level (e.g., incomplete retrieval of identified research, reporting bias).	25
Conclusions	26	Provide a general interpretation of the results in the context of other evidence, and implications for future research.	26
**Funding**			
Funding	27	Describe sources of funding for the systematic review and other support (e.g., supply of data); role of funders for the systematic review.	27

Helen A Harris-Fry *et al*. | Online Supplementary Material

Supplemental Table 2: Sample search string
Formatted for MEDLINE (Ovid)

1<br>((land adj5 right*) OR landownership OR (land adj5 own*) OR (land adj5 tenure) OR (property adj5 right*) OR (land adj5 tit*) OR (land adj3 use*) OR (asset* adj5 own*) OR (asset* adj5 right*) OR (livestock adj5 own*) OR (livestock adj5 right*) OR (land adj5 access) OR (property adj5 access) OR (poultry adj5 own*) OR (control* adj5 land) OR (control* adj5 asset*) OR (control* adj5 livestock) OR (inherit* adj5 right*) OR (inherit* adj5 practic*) OR (farmland adj5 right*) OR (farmland adj5 own*) OR (farmland adj5 tenure) OR (farmland adj5 tit*) OR (farmland adj5 access) OR (farmland adj5 control) OR (inherit* adj3 (asset* OR land OR farmland)) OR ((decision* OR power) adj3 (land OR livestock OR farmland OR asset* OR poultry))).ti,ab,kw,sh.
2<br>(wage* OR remunerat* OR pay* OR paid OR salar* OR income* OR labor* OR labour* OR employ* OR workforce OR ((vocation* OR job* OR occupation* OR profession*) adj3 (opportunit* OR participa*)) OR (livelihood* adj3 opportunit*) OR ((intrahousehold OR "intra household") adj5 bargain*) OR ((intrahousehold OR "intra household") adj5 allocat*) OR ((intrahousehold OR "intra household") adj5 (decision* adj3 (make* OR making OR made))) OR (within adj3 (household adj5 (allocation OR bargain*)))) .ti,ab,kw,sh.
3<br>(task* OR (time adj3 use*) OR activit* OR (time adj3 allocat*) OR workload* OR (energ* adj3 expend*)).ti,ab,kw,sh.
4<br>(nutr* OR nourish* OR undernutrition OR undernourish* OR underweight OR bmi OR body mass index OR anthropom* OR stunt* OR haz OR height-for-age OR length-for-age OR wasting OR wasted OR WHZ OR WLZ OR WAZ diet* OR mdds OR mdd-w OR mddw OR dds OR diversity score OR malnutrition OR caloric deficienc* OR malnourish* OR food security OR food insecurity OR (food adj5 expenditure*) OR (food adj5 consumption) OR (food adj3 share*) OR (staple* adj5 share) OR hfias OR ((food OR calorie) adj3 intake) OR ((intrahousehold OR intra household) adj3 welfare)).ti,ab,kw,sh.
5<br>(((sex OR gender* OR female* OR woman OR women) AND (equit* OR equalit* OR inequit* OR inequal* OR unequal* OR discriminat* OR power OR bargain* OR empower* OR disempower*)) OR ((sex OR gender* OR female* OR woman OR women) adj5 power)).ti,ab,kw,sh.
6<br>(((sex OR gender* OR female* OR woman OR women) adj3 (bias* OR parity)) OR (effects adj3 gender)).ti,ab,kw,sh.
7<br>((sex OR gender* OR female* OR woman OR women) AND ((share OR gap OR distrib*) adj3 (land OR landownership OR property OR asset* OR livestock OR poultry OR inherit* OR farmland OR wage* OR remunerat* OR pay* OR paid OR salar* OR income* OR labor* OR labour* OR employ* OR workforce OR ((vocation* OR job* OR occupation* OR profession*) adj3 (opportunit* OR participa*)) OR task* OR activit* OR workload*))).ti,ab,kw,sh.

Helen A Harris-Fry *et al*. | Online Supplementary Material

8<br>(((intrahousehold OR intra household) adj5 bargain*) OR ((intrahousehold OR intra household) adj5 allocat*) OR ((intrahousehold OR intra household) adj5 (decision* adj3 (make* OR making OR made))) OR (within adj3 (household adj5 (allocation OR bargain*))) AND (sex OR gender* OR female* OR woman OR women)).ti,ab,kw,sh.
9<br>(1 or 2 or 3) and 4 and (5 or 6 or 7 or 8)
10<br>(afghanistan OR albania OR algeria OR angola OR argentina OR armenia OR armenian OR azerbaijan OR bangladesh OR benin OR byelarus OR byelorussian OR belarus OR belorussian OR belorussia OR belize OR bhutan OR bolivia OR bosnia OR herzegovina OR hercegovina OR botswana OR brazil OR bulgaria OR Burkina Faso OR Burkina Fasso OR Upper Volta OR burundi OR urundi OR cambodia OR Khmer Republic OR kampuchea OR cameroon OR cameroons OR cameron OR camerons OR Cape Verde OR Central African Republic OR chad OR china OR colombia OR comoros OR comoro islands OR comores OR mayotte OR congo OR zaire OR Costa Rica OR Cote d'Ivoire OR Ivory Coast OR cuba OR djibouti OR somaliland OR dominica OR Dominican Republic OR East Timor OR East Timur OR Timor Leste OR ecuador OR egypt OR United Arab Republic OR El Salvador OR eritrea OR ethiopia OR fiji OR gabon OR Gabonese Republic OR gambia OR gaza OR Georgia Republic OR Georgian Republic OR ghana OR grenada OR guatemala OR guinea OR guiana OR guyana OR haiti OR honduras OR india OR maldives OR indonesia OR iran OR iraq OR jamaica OR jordan OR kazakhstan OR kazakh OR kenya OR kiribati OR korea OR kosovo OR kyrgyzstan OR kirghizia OR Kyrgyz Republic OR kirghiz OR kirgizstan OR Lao PDR OR laos OR lebanon OR lesotho OR basutoland OR liberia OR libya OR macedonia OR madagascar OR Malagasy Republic OR malaysia OR malaya OR malay OR sabah OR sarawak OR malawi OR mali OR Marshall Islands OR mauritania OR mauritius OR Agalega Islands OR mexico OR micronesia OR Middle East OR moldova OR moldovia OR moldovian OR mongolia OR montenegro OR morocco OR ifni OR mozambique OR myanmar OR myanma OR burma OR namibia OR nepal OR Netherlands Antilles OR nicaragua OR niger OR nigeria OR muscat OR pakistan OR palau OR palestine OR panama OR paraguay OR peru OR philippines OR philipines OR phillipines OR phillippines OR Papua New Guinea OR romania OR rumania OR roumania OR rwanda OR ruanda OR Saint Lucia OR St Lucia OR Saint Vincent OR St Vincent OR grenadines OR samoa OR Samoan Islands OR Navigator Island OR Navigator Islands OR Sao Tome OR senegal OR serbia OR montenegro OR seychelles OR Sierra Leone OR Sri Lanka OR Solomon Islands OR somalia OR sudan OR suriname OR surinam OR swaziland OR South Africa OR syria OR tajikistan OR tadzhikistan OR tadjikistan OR tadzhik OR tanzania OR thailand OR togo OR Togolese Republic OR tonga OR tunisia OR turkey OR turkmenistan OR turkmen OR uganda OR ukraine OR uzbekistan OR uzbek OR vanuatu OR New Hebrides OR venezuela OR vietnam OR Viet Nam OR West Bank OR yemen OR zambia OR zimbabwe).ti,ab,kw,sh.
11<br>(((developing OR less* developed OR under developed OR underdeveloped OR middle income OR low* income OR underserved OR under served OR deprived OR poor*) adj2 (countr* OR nation* OR population* OR world OR state*)) OR ((developing OR less* developed OR under developed OR underdeveloped OR middle income OR low* income) adj2 (economy OR economies)) OR (low* adj2 (gdp OR

	gnp OR gross domestic OR gross national)) OR (low adj3 middle adj3 countr*) OR (lmic OR lmics OR (third adj2 world) OR lami countr* OR transitional countr*) OR Africa OR (Africa* NOT African American) OR asia* OR Latin America* OR South America* OR Central America* OR caribbean OR oceania* OR Middle East* OR mena).ti,ab,kw,sh.
12	14 10 OR 11
13	9 AND 12

Helen A Harris-Fry *et al*. | Online Supplementary Material

Supplemental Table 3: Databases, repositories and journals

**Databases**	• EBSCO<br>• Medline<br>• Scopus<br>• Web of Science<br>• Popline<br>• CAB<br>• Eldis<br>• OpenTrial<br>• Bridge Data<br>• AGRIS (including FAO and IFPRI libraries, listed separately in the protocol)
**Hand-searched repositories**	• Bill and Melinda Gates Foundation Grants Database<br>• DfID Research for Development Outputs<br>• 3ie Impact Evaluation Repository<br>• The World Bank IEG evaluations<br>• UNICEF Evaluation Database<br>• USAID DEC<br>• AEA RCT Registry<br>• Cochrane Library
**Journals**	• American Journal of Agricultural Economics<br>• Food Policy<br>• Journal of Development Economics<br>• Maternal and Child Health Journal<br>• Public Health Nutrition<br>• The Journal of Development Studies<br>• The Journal of Nutrition<br>• The Lancet<br>• The Lancet Global Health<br>• The proceedings of the Argiculture, Nutrition and Health Academy conference<br>• The proceedings of the CSAE Conference<br>• The proceedings of the NEUDC Conference<br>• The World Bank Economic Review<br>• World Development

Helen A Harris-Fry *et al*. | Online Supplementary Material

Supplemental Table 4: Characteristics of included studies

	**Country**	**Sample included in analysis**	**Data type**	**Exposure(s)**	**Outcome(s)**
**QUANTITATIVE STUDIES**					
**Gender inequity in income (*n*=15)**					
Razzaque & Toufique (2007) (24)	Bangladesh	1038 households	Cross-sectional survey	Women's share of household income	Food share
Gaiha & Kulkarni (2005) (25)	India	26 854 households	Cross-sectional survey	Male-female wage difference	Severe stunting (height-for-age < -3 SD)
Lancaster, Maitra & Ray (2006) (26)	India (rural Kerala)<br>India (rural Bihar)<br>India (rural Maharashtra)	1321 households<br>2824 households<br>2131 households	Cross-sectional survey	Women's share of household wage income	Food share
Senauer & Garcia (1988) (27)	Philippines	140 households; 476 individuals<br>~800 households; 2320 observations	Longitudinal survey; 4 waves	Mother's wage vs. father's	Child's energy adequacy ratio<br>Height-for-age z-score<br>Weight-for-height z-score
Hopkins, Levin & Haddad (1994) (23)	Niger	452 observations; 135 households	Longitudinal survey	Women's income vs. men's	Ln food expenditure
Aromolaran (2004) (28)	Nigeria	2573 individuals; 472 households	Fortnightly measurements, treated as a cross-section in analysis	Women's share of household income	Ln food expenditure
Hoddinott & Haddad (1994) (10)	Côte d'Ivoire	1503 households	Cross-sectional survey	Wives' share of household cash income	Ln food expenditure
Duflo & Udry (2004) (29)	Côte d'Ivoire	973 observations; > 800 households	Longitudinal survey; 3 waves	Change in women's income vs. change in men's	Ln food expenditure
Van den Broeck, Van Hoyweghen, & Maertens (2018)(30)	Senegal	461 households	Longitudinal survey; 2 waves	Women's employment in agricultural export sector vs. men's	Household Food Insecurity Assess Scale
Lachaud (1998)(31)	Burkina Faso	1352 households	Cross-sectional survey	Women's share of household income	Underweight (WAZ < -2 SD)<br>Stunting (HAZ < -2 SD)<br>Wasting (WHZ < -2 SD)
		4744 households	Cross-sectional survey	Women's share of household income	Food share

	Country	Sample included in analysis	Data type	Exposure(s)	Outcome(s)
Marinda (2006)(32)	Kenya	129 households	Cross-sectional survey	Mothers' income minus men's	Height-for-age z-score
Shoo (2011)(33)	Tanzania	152 households	Cross-sectional survey	Mother has a non-farming source of income vs. father	Weight-for-age z-score
Josephson (2018) (34)	Malawi	693 households	Longitudinal survey; 2 waves	Change in women's Ln agricultural income vs. men's	Ln food expenditure
McCarthy & Kilic (2017) (35)	Malawi	1929 households; 3858 observations	Longitudinal survey; 2 waves	Women earn all unpooled income vs. men earn all	Food expenditure Food share
Attanasio & Lechene (2002) (45)	Mexico	7742 households	Longitudinal survey; 5 waves	Women's share of household income	Food share
**Gender inequity in land and livestock (*n*=10)**					
Santos et al. (2014) (36)	India	1035 households	Longitudinal survey; 2 waves	Women's name on land title vs. men's	Household Dietary Diversity Score
Menon, Van der Meulen Rodgers & Nguyen (2014) (37)	Vietnam	7623 individuals in 2004 and 7203 individuals in 2008; 1728 households	Longitudinal survey; 2 waves	Women have land use certificate vs. men	Food share
Quisumbing & Maluccio (2003) (38)	Indonesia	114 households	Cross-sectional survey	Ln wife's land size at marriage vs. husband's, in hectares	Food share
Pangaribowo (2013) (39)	Indonesia	Not reported	Longitudinal survey	Women's share of household livestock assets	Food share
Kusago & Barham (2001)(50)	Malaysia	120 households	Cross-sectional survey	Women's share of household assets (predominantly land)	Food share
Muchomba (2017) (41)	Ethiopia	1061 households	Longitudinal survey; 7 waves	Joint land titling vs. men only	Food share<br>Ln food expenditure
Kumar (1994) (42)	Zambia	213 households	Longitudinal survey	Share of household land size farmed by women (jointly or individually)	Ln household dietary diversity score
Doss (2006) (43)	Ghana	Not reported	Repeated cross-sectional survey	Women's share of household agricultural land	Food share
Quisumbing & Maluccio (2003) (38)	Ethiopia	1347 households	Cross-sectional survey	Ln value of wife's land and livestock at marriage vs. husband's, in Ethiopian Birr	Food share
Jin & Iannotti (2014) (44)	Kenya	183 individuals	Cross-sectional	Women's livestock value	Height-for-age z-score

Helen A Harris-Fry *et al*. | Online Supplementary Material

	Country	Sample included in analysis	Data type	Exposure(s)	Outcome(s)
			survey	(solely or jointly owned) minus men's (solely owned), in Kenyan Shillings	Weight-for-age z-score Weight-for-height z-score
**Gender inequity in work burdens (*n*=1)**					
Kumar (1994) (42)	Zambia	213 households	Longitudinal survey	Male-female difference in predicted annual household maintenance labour hours	Ln household dietary diversity score
**QUALITATIVE STUDIES**					
Chaturvedi, et al. (2016) (46)	India	647 (509 individual interviews + 66 groups + 72 non-formal interactions)	Interviews, focus group discussions, non-formal interactions	Inequity in time burden	Child dietary quality and undernutrition
Nichols (2016) (47)	India	81 individuals	Interviews supported by participant observation	Inequity in workload	Women's dietary quality
Morrison et al. (2017) (48)	Nepal	25 interviews + 2 groups	Interviews and focus group discussions	Inequity in earned income<br>Inequity in workload	Women's dietary quality
Mwangome et al. (2010) (49)	Gambia	63 individuals (8 groups)	Focus group discussions	Inequity in workload	Child dietary quality
Me-Nsope, et al. (2016) (50)	Malawi	329 individuals (39 key informants and 24 groups)	Key informant and group interviews	Inequity in land inheritance<br>Inequity in income	Household food security
Geheb et al. (2008) (51)	Kenya, Uganda, Tanzania	254 individuals	Focus group discussions, informal interviews, observations	Inequity in workforce participation	Household food expenditures
Galiè et al. (2015) (52)	Tanzania	105 individuals	Participatory interviews	Inequity in income<br>Inequity in livestock	Household food security
	Ethiopia	18 individuals	Participatory interviews	Inequity in livestock<br>Inequity in labour norms	Household food security
	Nicaragua	12 individuals	Participatory interviews	Inequity in income	Household food security

Helen A Harris-Fry *et al.* | Online Supplementary Material

Supplemental Table 5: Risk of bias assessment for quantitative studies, using an adapted version of the ROBINS-I tool

	Confounding	Selection	Exposure classification	Missing data	Outcome measurement	Reporting	Instrumentati on	Overall
**Gender inequity in income**								
Razzaque & Toufique	critical	no information	moderate	no information	moderate	moderate	critical	critical
Gaiha & Kulkarni	serious	low	moderate	serious	low	serious	critical	critical
Lancaster, Maitra & Ray	moderate	low	low	moderate	moderate	serious	serious	serious
Senauer & Garcia (energy adequacy ratio)	critical	serious	serious	no information	low	moderate	critical	critical
Senauer & Garcia (height-for-age)	critical	serious	serious	no information	low	moderate	critical	critical
Senauer & Garcia (weight-for-height)	critical	serious	serious	no information	low	moderate	critical	critical
Hopkins, Levin & Haddad	serious	moderate	serious	no information	low	moderate	critical	critical
Aromolaran	serious	moderate	serious	low	serious	moderate	serious	serious
Hoddinott & Haddad	serious	low	serious	low	moderate	moderate	serious	serious
Duflo & Udry	moderate	low	moderate	moderate	low	moderate	moderate	moderate
Van den Broeck, Van Hoyweghen, & Maertens	moderate	moderate	moderate	low	moderate	moderate	not applicable	moderate
Lachaud (food share)	critical	low	serious	low	moderate	moderate	critical	critical
Lachaud (low height-for-age)	critical	low	serious	low	moderate	moderate	critical	critical
Lachaud (low weight-for-height)	critical	low	serious	low	moderate	moderate	critical	critical
Lachaud (low weight-for-age)	critical	low	serious	low	moderate	moderate	critical	critical
Marinda	critical	moderate	no information	low	low	serious	no information	critical
Shoo	critical	moderate	moderate	serious	moderate	serious	not applicable	critical
Josephson	serious	serious	critical	critical	low	moderate	critical	critical
McCarthy & Kilic	serious	low	critical	low	moderate	moderate	not applicable	critical
Attanasio & Lechene	moderate	low	moderate	moderate	low	moderate	moderate	moderate
**Gender inequity in land and livestock**								
Santos et al.	critical	moderate	low	serious	low	moderate	not applicable	critical
Menon, Van der Meulen Rodgers & Nguyen	moderate	moderate	moderate	moderate	low	moderate	not applicable	moderate
Quisumbing & Maluccio	serious	low	moderate	low	low	moderate	critical	critical
Pangaribowo	critical	low	moderate	no information	low	moderate	not applicable	critical
Kusago & Barham	serious	serious	no information	no information	moderate	moderate	not applicable	critical
Muchomba (food share)	critical	moderate	serious	low	low	moderate	not applicable	critical
Muchomba (food expenditure)	serious	moderate	serious	low	low	moderate	not applicable	serious
Kumar	critical	low	low	critical	moderate	moderate	not applicable	critical
Doss (1992)	critical	low	moderate	no information	low	moderate	not applicable	critical
Doss (1999)	critical	low	moderate	no information	low	moderate	not applicable	critical
Quisumbing & Maluccio	serious	low	moderate	low	low	moderate	critical	critical
Jin & Iannotti (height-for-age z-score)	critical	no information	moderate	no information	low	moderate	not applicable	critical
Jin & Iannotti (weight-for-height z-score)	critical	no information	moderate	no information	low	moderate	not applicable	critical
Jin & Iannotti (weight-for-age z-score)	critical	no information	moderate	no information	low	moderate	not applicable	critical
**Gender inequity in workloads**								
Kumar	critical	low	no information	critical	moderate	moderate	no information	critical

critical; serious; moderate; low; no information; not applicable

Helen A Harris-Fry *et al.* | Online Supplementary Material

Supplemental Table 6: Quality assessment of qualitative studies, using Lockwood, Munn and Porritt tool

	South Asia			Sub-Saharan Africa			Multiple regions*
Quality domain from Lockwood, Munn & Porritt	Chaturvedi et al.	Nichols	Morrison et al.	Geheb et al.	Mwangome et al.	Me-Nsope et al.	Galiè et al.
1. Is there congruity between philosophical perspective and methodology?	Y	PY	PY	N	Y	PY	Y
2. Is there congruity between research question and methodology?	Y	PY	Y	PY	PY	PY	Y
3. Is there congruity between methodology and sampling methods?	PY	PY	Y	PY	N	NI	PN
4. Is there congruity between methodology and methods used to collect data?	Y	NI	Y	NI	PY	PY	PY
5. Is there congruity between methodology and representation and analysis of data?	PY	NI	Y	NI	PN	N	PN
6. Is there congruity between methodology and interpretation of results?	PY	N	PY	PN	PN	N	Y
7. Is there a statement locating researcher culturally or theoretically?	N	PN	N	N	PY	N	N
8. Is the influence of the researcher on the research, and vice-versa, addressed?	PN	N	PN	N	N	N	N
9. Are participants, and their voices, adequately represented?	Y	PY	Y	NI	PY	PN	PY
10. Is the research ethical according to current criteria and is there evidence of ethical approval by an appropriate body?	Y	NI	Y	NI	Y	PN	Y
11. Do the conclusions follow from the analysis, or interpretation, of the data?	Y	NI	Y	N	PY	PN	Y
Overall quality appraisal	High	Low	High	Critical	Low	Low	Medium

Y=Yes; PY=Probably Yes; PY=Probably No, N=No; NI=No information.
